# The complete chloroplast genome of *Elaeocarpus japonicus* Sieb. et Zucc. (Elaeocarpaceae)

**DOI:** 10.1080/23802359.2021.1872454

**Published:** 2021-02-12

**Authors:** Yihui Wang, Zhixiang Zhang, Yifei Xie

**Affiliations:** a School of Life Sciences, Gannan Normal University, Ganzhou, P.R. China; b School of Ecology and Nature conservation, Beijing Forestry University, Beijing, P.R. China

**Keywords:** *Elaeocarpus japonicus*, chloroplast genome, illumina sequencing, phylogenetic analysis

## Abstract

The complete chloroplast genome of *Elaeocarpus japonicus* Sieb. et Zucc. was reported in this study. The cp genome was 157,639 bp in length including two inverted repeats (IRs) of 27,437 bp, which were separated by large single copy (LSC) and small single copy (SSC) of 85,784 bp and 16,981 bp, respectively. The GC content was 37.2%. The genome encoded 111 functional genes, including 77 protein-coding genes, 30 tRNA genes, and 4 rRNA genes. The phylogenetic tree showed that *E. japonicus* representing Elaeocarpaceae is sister to *Averrhoa carambola* representing Oxalidaceae with strong support.

*Elaeocarpus* is the most species-rich genus in Elaeocarpaceae (Oxalidales), comprising 350-400 species of trees that grows in tropical and subtropical forests (Phoon 2015). There are 39 species and 6 varieties in China, which mainly distributed in South and Southwest China (Zhang 1989; Tang and Phengklai 2013; Xie 2018).

The object of this study is a widespread evergreen tree, *Elaeocarpus japonicus* Sieb. et Zucc., which is a useful medicinal species as its dried bark decoction can treat kidney and duodenal diseases in Japan (Higashi et al. 1976). The leaf materials of *E. japonicus* were collected from the field at Wugong Mountain, Jiangxi Province (N 27°27'39″, E 114°11'8″) and immediately dried by silica gel for DNA extraction. Voucher specimen (*Renlin LIU*160730004,20160730) of the collection was deposited at Museum of Beijing Forestry University (BJFC). Total genomic DNA was extracted using the magnetic beads method and then sent to Sino Geno Max company for next generation sequencing using Illumina HiSeq (TM) 2000 in Beijing, China. After removing the low quality reads from the raw data, we got the clean data of 15.28GB, named DMS14628-s.fq, and uploaded it to SRA database of NCBI (PRJN660422, SRR12574443, SAMN15949251) with fastq format. We used Geneious 11.0 to assemble and Plastid Genome Annotator to annotate taking published plastid genome of Brunellia trianae (MN585217) as reference (Kearse et al. 2012). The cp genome of *E. japonicus* was 157,639 bp in length (MT985378) and the GC content was 37.2%. LSC and SSC contained 85,784 bp and 16,981 bp, respectively, while IR was 27,437 bp in length. The chloroplast genome encoded 111 functional genes, including 77 protein-coding genes, 30 tRNA genes, and 4 rRNA genes.

The phylogenetic tree, containing the complete chloroplast genomes of 47 species, was reconstructed based on the maximum-likelihood by MEGA7.0 software (Kumar et al. 2016). The results showed that *E. japonicus* representing Elaeocarpaceae is sister group to *Averrhoa carambola* (Oxalidaceae) with strong support (Figure 1). It is obvious that Elaeocarpaceae should be placed in Oxalidales, which is congruence with the previous studies of Savolainen et al. (2000).

**Figure 1.**
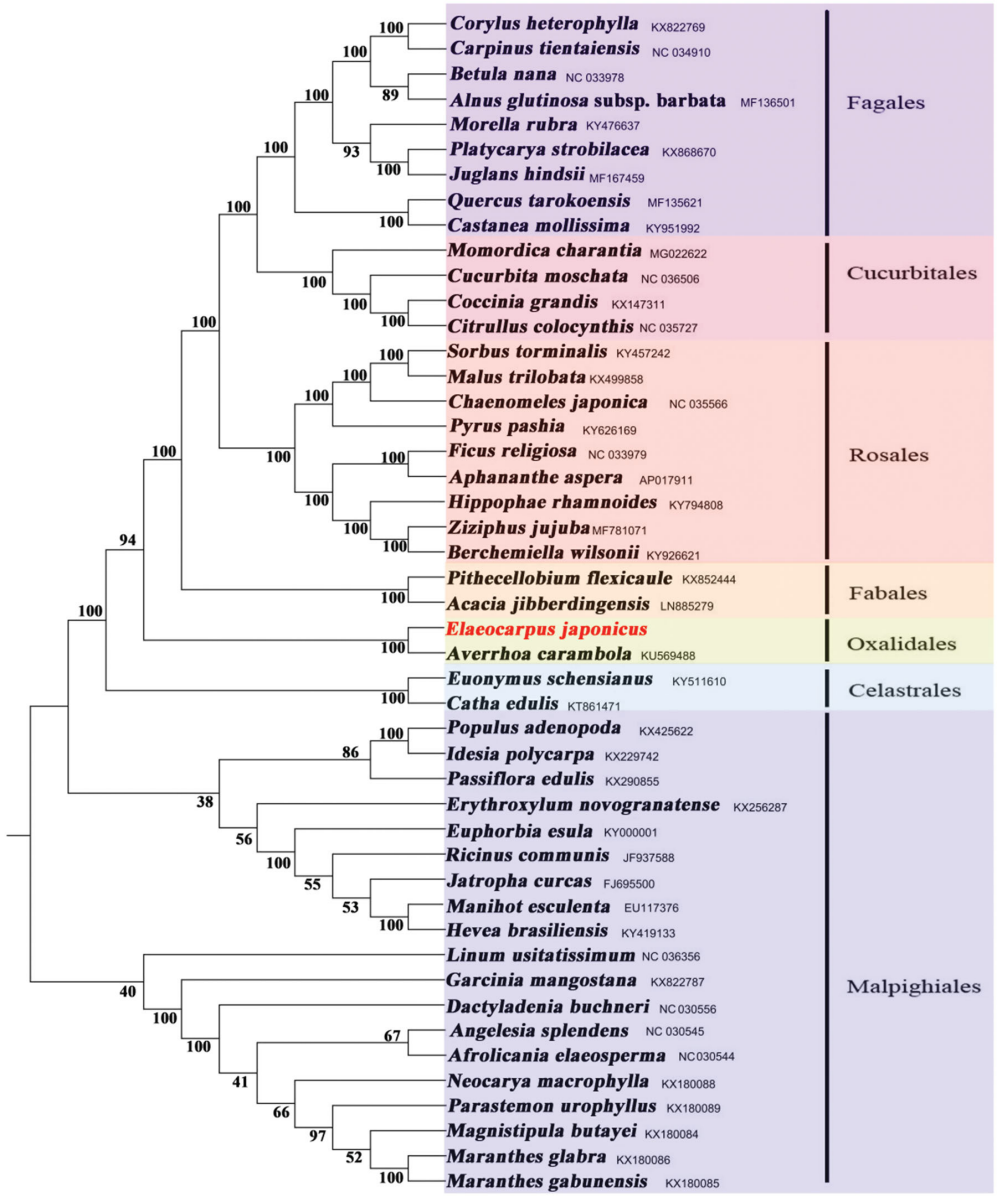
A phylogenetic tree of Fabids, based on the maximum-likelihood method constructed using the complete chloroplast genomes of *Elaeocarpus japonicus* and 46 other species.

## Data Availability

The data that support the findings of this study are available in GenBank, reference number (MT985378). These data were derived from the following resources available in the public domain: (constructed phylogenetic tree: KX822769, NC034910, NC033978, MF136501, KY476637, KX868670, MF167459, MF135621, KY951992, MG022622, NC036506, KX147311, NC035727, KY457242, KX499858, NC035566, KY626169, NC033979, AP017911, KY794808, MF781071, KY926621, KX852444, LN885279, KU569488, KY511610, KT861471, KX425622, KX229742, KX290855, KX256287, KY0000001, JF937588, FJ695500, EU117376, KY419133, NC036356, KX822787, NC030556, NC030545, NC030544, KX180088, KX180089, KX180084, KX180086, KX180085, https://www.ncbi.nlm.nih.gov/genbank/)
